# Medical Opinion and Movement

**Published:** 1909-03-13

**Authors:** 


### Medical Opinion and Movement.

The much-discussed question of how to avoid leaving sponges and other foreign bodies in the abdominal cavity during laparotomy is exhaustively considered in the *American Journal of Obstetrics* by Dr. Crossen. This author considers, and in turn condemns as fallible, every method so far suggested. He quotes the case of one surgeon who was so determined to avoid the accident that he used only swabs to which a long tape was knotted whose other end was gripped in an artery clip. Unfortunately one day the whole contrivance, clip and tape and gauze, was left in the abdomen. He also mentions that at an American hospital a rule was made that nothing shorter than six-foot lengths of gauze were to be used as swabs. Even one of these got lost and shut up without its absence being detected. His own reflections lead him to the conclusion that one very important precaution is to employ no small or short instruments during laparotomy. All forceps, clips, needle-holders, and other instruments should be at the least six and a half inches long, and better, eight inches. As regards the sponges, he has adopted a system of gauze rolls twelve feet in length, one end of which is sewn into a pocket on the sterilised sheet. By an ingenious arrangement the sterilisation is rendered very simple, and all chances of contamination avoided. It will certainly be astonishing if this operator ever leaves a swab in the abdomen. But it is to be noted that by his system he cuts himself off from the use of the hot cloths which most surgeons believe to be valuable whenever the intestines have to be much exposed or handled.

Among many details concerning the diagnosis and treatment of syphilis contributed to the *Dublin Journal of Medical Science* by Dr. J. J. Abraham, the experience of this author at the Lock Hospital of the use of arsenic in the forms of soamin and orsudan are of interest. Twenty female patients underwent treatment by injection of ten grains twice a week for five weeks. It was found that the injections are painless and are not followed by sepsis, and that symptoms of arsenical poisoning never appeared. Notwithstanding the repute of arsenic among dermatologists, the rash did not disappear in a single case. The author observes that as the syphilitic roseola has a well-known tendency to disappear spontaneously, the drug can hardly be regarded as having the very slightest effect in this respect. Further, in several cases fresh mucous plaques and condylomata appeared while the patients were under treatment; some condylomata healed, others remained *in statu quo*. In no case can it be said that the symptoms of the disease disappeared during the course. Knowing the effects of mercury in like cases, it was concluded (reluctantly, Dr. Abraham says, though it is a little difficult to see why the results of a research should be so regarded) that arsenic in these forms is undoubtedly inferior to mercury. It is also to be noted that the doses given were but two-thirds of those suggested by Colonel Lambkin. This was fixed because of the smaller body weight of women as compared with men, and probably in no way invalidates the results. It is also noteworthy that according to quite recent researches soamin does not differ, as it was supposed to do, from atoxyl. The formulæ are known to be the same, but a variation in the molecular arrangement was believed to exist which has now been disproved.

Merycism, or rumination, in man was considered two years ago in a paper published by Dr. E. M. Brockbank, who now gives in the *Medical Chronicle* four more cases of the same habit. The common factors of these patients seem to be mainly as follows. The process begins either in youth or early in adult life. It is not unpleasant, being unattended by nausea or discomfort. There is no instinctive desire to spit out the regurgitated food, but rather to rechew it and reswallow it. It is not a symptom of an overloading of the stomach, because in one instance the point was tested by spitting out everything thus brought up for rumination, and the result was rapid emaciation. It appears that a certain amount of selection is exercised by the stomach as to what it will reject; but this varies in different individuals. Sometimes it is the particularly indigestible, such as orange pips, stringy meat, or insufficiently masticated food. It is also interesting to note that particular liquids are sometimes ruminated, if the term can be allowed. One man thus returns Benedictine, another one claret and port but not champagne. Sometimes the act is entirely unconscious, sometimes a kind of effort is made in response to a definite sensation of gastric tension. All the ruminants seem quite pleased with the possession of this peculiar power, and have no wish to undergo treatment for its abolition. Only one of the four gives any family history of a similar arrangement of the gastric and masticatory functions. In that case a sister was also the subject of merycism.

In private practice the question of the treatment of obesity not infrequently arises, and there are so many styles of diet scales for this condition that the medical attendant may well be pardoned for wavering between the different tables that are presented to his notice. In practice he has to judge the merit of the different cures by their results; and not infrequently by the endurance and the energy of his patients. One of the most irksome restrictions which conventional—if such an adjective may be applied to strictly professional subjects—cures enforce is abstention from liquids. Schweninger first put forward the claim that a limitation of the intake of liquids is an essential in the treatment of obesity, on the ground that metabolic processes are much increased in thirsty subjects. This argument was accepted without much comment, until Salomon showed that it is no argument at all, as Schweninger's facts can not be substantiated. Metabolic processes in a thirsty patient are not more active than in one who takes liquids freely, nor is it a fact, as Schweninger alleged, that dry meals are less easily absorbed than moist ones. Richter, discussing modern obesity cures, points out that a diet that will satisfy the patient without adding to his weight is the most desirable. He favours potatoes as the principal article of diet. A potato diet easily satisfies, as the amount to be taken in order to still the cravings of appetite is much smaller than is the case with an article of food such as meat, whose dietetic value is far higher, and it is easily digestible and capable of being "exhibited" in an endless variety of forms. At the same time, he allows a large amount of vegetables, provided they are simply boiled or given raw in the shape of salads prepared without oil. Fruit, especially acid fruit, is permissible, and a valuable drink is ordinary lemonade, made of fresh lemons, without addition of sugar.

---

With such diet, the patient's cravings, so distressing a feature with the ordinary starvation cures, are not marked, especially if the menu is varied day by day, and small quantities of meat given once a day. Bread is allowed only in the smallest possible amount, and then always in the form of toast. Where, as is often the case with fat patients, there is a slight amount of sugar in the urine, this diet tends to increase the glycosuria, and may therefore have to be modified. So also in cases of marked indigestion, which a diet of potatoes is apt to make worse. Yet another objection to the prescribed diet is the fact that it tends to constipate the patient. This tendency must be counteracted by the administration of raw vegetables and fruit, aided, when needful, by a wineglassful or more of some mild aperient. The author lays stress on the concomitant treatment. Patients should be advised to live in a cold climate, preferably in a mountainous region, and to take a moderate amount of exercise. Massage is a valuable auxiliary in the treatment, but only when combined with active exercise. Bathing and cold-water cures do not effect much, in the author's opinion, while thyroid and ovarian extract are inadmissible in nearly all cases.

---

The difficulty of making an early diagnosis of pulmonary tuberculosis is well recognised in cases in which there is no cough, and consequently no expectoration. Dr. Th. Hausmann claims to have discovered several times signs of early tuberculosis at the apex of the lungs in patients who have consulted him for dyspeptic troubles, and it has occurred to him that the diagnosis might be confirmed by examining the gastric contents in the morning while fasting, for sputum collecting in the larynx and swallowed instead of being expectorated. In the first case in which he put this idea into practice he obtained a positive result. The patient was a man complaining of dyspeptic symptoms with anorexia and general weakness. He had no cough or expectoration, and several physicians had variously diagnosed anæmia, neurasthenia, and gastric catarrh. Under the right clavicle however the author discovered a slight dulness with fine crepitation on deep inspiration. By gastric lavage in the morning he obtained a small lump of mucus resembling sputum, and on examination this was found to contain alveolar cells and a few bacilli of Koch. Since then Dr. Hausmann has been able to confirm the diagnosis of pulmonary tuberculosis in six other cases by the same method. Absence of expectoration has been generally supposed to justify the assumption of a "closed" tubercular lesion, as distinguished from an open lesion with expectoration; but these facts would tend to show that the absence of expectoration does not justify such a conclusion.

---

Although the therapeutic value of the Röntgen rays and other radiations of a similar nature is now beyond question for quite a number and variety of affections, the mechanism underlying the curative process is by no means clearly demonstrated. The earliest applications of the *x*-rays were made on malignant growths, and the diminution in size of the growths under the influence of the radiations led to the supposition that the rays exercised a special destructive effect upon the cells of the growth. This necrosing action of the rays was also manifested on healthy tissue when the dose administered was beyond a certain strength. Moreover, histological examination of tumours so treated have shown the cancer cells in process of destruction, with changes in their form and structure, and have confirmed radiologists in this view. In a very interesting and suggestive contribution to the *Journal de Médecine*, Dr. L. Raoult-Deslongchamps suggests that the curative property of *x*-rays lies in their power to excite leucocytosis. Histological examination of animal tissues after exposure to the rays have shown, according to this author a definite leucocytic infiltration, and the abundant secretions from open tumours after treatment with the rays have been found to contain large quantities of leucocytes. He points out that the good results obtained in leukæmia, adenopathies, varicose ulcers, chronic eczemas, etc., find a more easy explanation on this hypothesis than on the ground of a specific destructive effect of the rays.

---

Moreover this hypothesis would appear to bring the *x*-rays into line with other physical agents in their therapeutic effects, such as light, heat, hot air, high frequency sparks, and the congestive method of Bier, all of which may be said to act by producing a local congestion, an increased flow of blood to the part, and at the same time a more or less intense leucocytosis. He is thus led to the rather broad, though enticing, generalisation that the underlying principle of all these therapeutic measures lies not in their direct effect upon the malady but in their ability to arouse the defensive powers of the organism itself. In regard to cancer it may be remarked that the disease appears to be particularly amenable to the action of the rays in such regions as the face, where the blood supply is abundant and congestion and leucocytosis can be readily induced. From this hypothesis the author deducts certain important conclusions in regard to radiotherapy. He thinks that all affections in which a local reaction is called for (arthritis, localised tuberculosis, cutaneous affections, neuritis, etc.) should be favourably influenced by the application of the *x*-rays, and that the field of radiotherapy should therefore be considerably widened. The success of the treatment must depend to some extent upon the general condition of the patient and his ability to respond to the leucocytic excitation, and the method of application must be adjusted in like measure. The idea that the maximum of radiation should be administered compatible with the integrity of the tissues is wrong. It must be just sufficient to call forth the requisite leucocytic reaction without exhausting the powers of the organism. On these grounds the author advocates frequent small doses, and emphasises the point that the treatment should not cease suddenly, but gradually, by smaller and less frequent applications.

---

Little is known in this country of so-called Kinesitherapy in the treatment of chronic affections of the uterus and its appendages. It owes its origin to Sweden the home of all forms of massage and exercise, where it has been practised by leading gynæcologists for many years, and more recently it appears to have gained favour in France and Germany. Briefly, the treatment consists in massage of the abdomen, combined with gymnastic exercises, in which all the muscles around the pelvis are brought into action. These exercises are carried out before and after the massage. The massage employed is chiefly circular friction with the pulp of the fingers over the abdomen, so that the abdominal skin glides over the subjacent tissues and organs, and vibration. The massage should be light and short, not more than three to five minutes. The method is described in a contribution by Dr. Gordon Block to the *Journal de Médécine*. He adopts the view that without denying the rôle played by micro-organisms in gynæcological affections, a large proportion of them are of a congestive nature, and their pathology is to be explained on these lines. A chronic congestion of the pelvic organs leads to infiltration of the ligaments and appendages, displacements and fixation of the uterus, and such functional derangements as menorrhagia, metrorrhagia, amenorrhœa, and the like. It is in the cure of these affections that the author claims remarkable results by the method of kinesitherapy. Like most of these physical therapeutic measures, it requires, if not any special contrivances, at least considerable skill and an expenditure of a large amount of time both by the operator and by the patient, and is therefore necessarily wearisome and expensive in its application. In fact the author insists that the treatment should be only carried out by the medical attendant himself.

---

The surgical treatment of optic neuritis is discussed at length by Dr. P. J. Hay in the *Ophthalmoscope*, and, also, less fully, by Dr. A. W. Stirling. In arrest of too hasty generalisations, the latter publishes details of nine cases seen in private in which expectant or medicinal treatment restored amblyopic eyes to perfect function: in some of these there was definite gross papillitis, in others little or no change in the fundus. Dr. Hay starts with the estimate that more than 50 per cent. of patients with optic neuritis and "choked disc" lose altogether the sight of the affected eye. Last year Sir Victor Horsley took up the position that optic neuritis is produced by a combination of factors, of which the only one certainly known to be present is a rise of intracranial tension; and he holds that all cases of optic neuritis should be relieved as soon as possible of such tension by operation, which should, in the absence of other indications consist in opening the subdural space in the temporal or subtentorial region. Dr. Hay points out that the difficulty is to ensure a permanent reduction of tension on account of the fact that only about 10 per cent. of intracranial tumours are completely removable. Tapping the sheath of the optic nerve was first suggested and performed by De Wecker 30 years ago, but the benefits proved so transient and uncertain that it was soon abandoned. Lumbar puncture is another operation easily performed with the same end of relieving tension. It has been found useful as a palliative measure in tuberculous meningitis, and also in other forms of meningitis. Its relative advantages compared with those of craniectomy are still the subject of diversity of opinion. If trephining is undertaken it is important that it should be done over the seat of the lesion, otherwise its effect will be much less. Paton has lately cast doubt upon the help afforded by the discs in diagnosing this, and probably the other signs are more reliable. The operation is better done early than late, and the prognosis as regards vision is fairly good in the former case.

---

According to an official statement in the *British Medical Journal*, his Majesty the King has consented to become the patron of the Radium Institute. A site has been acquired in Riding House Street, Portland Place, and the building will be erected there with as little delay as possible. The architect is Mr. T. P. Figgis, F.R.I.B.A. The Institute, according to the same authority, will be conducted so far as its general features are concerned upon the lines of the Radium Institute in Paris. In addition to the superintendent, the assistant to the superintendent, and the director of the laboratory, there will be an honorary medical and surgical staff, the members of which have yet to be appointed. The Institute hopes to acquire radium to the amount of 5 grams. The treatment carried out will be strictly limited to measures involving the use of radium or other radio-active substances. Treatment of cases by the Röntgen rays, the Finsen light, and by electrical currents will have no place in the Institute, as such measures of treatment are rightly considered by the organisers of the Institute to be already very amply provided elsewhere. The building will be in two parts, with separate entrances: one section for necessitous patients and the other for the well-to-do. The former will be treated free; the latter will be required to pay fees on a scale to be determined by the medical and surgical staff. Demonstrations in the use of radium will be given, and medical practitioners will be able to receive advice on the mode of employment of radium and information as to the radio-activity of their own specimens of radium.

